# Comparative genomic signatures in young and old Chinese patients with colorectal cancer

**DOI:** 10.1002/cam4.3987

**Published:** 2021-05-26

**Authors:** Fei Wang, Huanqing Cheng, Xiao Zhang, Lina Shan, Bingjun Bai, Kangke Chen, Feng lou, Shanbo Cao, Huina Wang, Sheng Dai

**Affiliations:** ^1^ Division of Colorectal Surgery Sir Run Run Shaw Hospital Zhejiang University School of Medicine Hangzhou China; ^2^ Prenatal Diagnosis Center Affiliated Hospital of Weifang Medical University Weifang China; ^3^ Division of Obstetrics and Gynecology Sir Run Run Shaw Hospital Zhejiang University School of Medicine Hangzhou China; ^4^ Zhejiang Province Key Laboratory of Biological Treatment Hangzhou China; ^5^ Division of Medicine Acornmed Biotechnology Co., Ltd. Beijing China

**Keywords:** Chinese, colorectal cancer, genomic landscape, old, young

## Abstract

**Background:**

Occurrence at a young age is known to be associated with unique clinical features in colorectal cancer (CRC). However, the genomic differences between young and old patients with CRC are not well elucidated and, to the best of our knowledge, have never been investigated in a Chinese population.

**Methods:**

Tumor tissue samples from 29 young (age ≤50 years) and 46 old (age >50 years) patients with CRC were collected. Targeted sequencing of 808 cancer‐related genes was conducted to characterize the genomic landscape for Chinese CRC.

**Results:**

Overall, mutational profiles exhibited notable differences between the two groups. In particular, *APC* and *PIK3CA* mutations were more frequently observed in old patients (*p* = 0.009 and *p* = 0.012, respectively), while *SMAD4* mutations tended to occur in young patients (*p* = 0.054). Mutation loci distributions of *KRAS* in the young cohort differed from those in the old cohort, and a higher frequency of *KRAS* codon 12 mutations was potentially associated with a young age (*p* = 0.076). The frequencies of clinically actionable alterations were analyzed by defined age categories, which unveiled a distinctive targeted genomic profile in the young group. Furthermore, among patients with mismatch repair‐proficient (pMMR) CRC, tumor mutation burden (TMB) was positively correlated with age (Pearson's *r* = 0.306, *p* = 0.011), and genomic alterations associated with high TMB in young patients differentiated from those in old patients.

**Conclusions:**

These findings revealed different molecular characterization between young and old Chinese patients with CRC, which may provide novel insights for the personalized treatment of CRC.

## Graphical abstract

This is the first NGS study to compare genomic profiles between young and old Chinese cohorts with CRC. The results demonstrated that young patients with CRC presented with some unique molecular features. Moreover, the comprehensive investigation of clinically actionable mutations and TMB will contribute to the improvement of personalized therapy and clinical management in CRC.

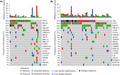

## 1 INTRODUCTION

Colorectal cancer (CRC) is the third most common type of cancer worldwide, with an estimated 1.8 million new cases and 800,000 deaths reported in 2018.^1^ CRC is generally considered to be a disease that occurs in patients >50 years, and young patients only account for a small proportion of all the patients with CRC.^2^ Although the incidence and mortality rate for patients >50 years of age have declined due to preventive screening, accumulating evidence indicates that CRC incidence is rising in the population of individuals <50 years of age.^3^, ^4^ The increasing prevalence of CRC among young individuals has led to a heavy burden on patients, their families, and society.

Patients with early‐onset CRC are more likely to exhibit distinctive biological behaviors, such as distal colon cancer and rectal cancer, low‐grade of differentiation, advanced stage, and development of metastatic disease.^5^, ^6^, ^7^, ^8^ Multiple studies have reported that patients diagnosed with CRC at <50 years old harbor a poor prognosis compared with old patients.^9^, ^10^ From the molecular perspective, the pathogenesis of early‐onset CRC is well characterized in patients with inherited CRC syndromes, in which some germline mutations of cancer susceptibility genes are identified.^11^, ^12^, ^13^, ^14^, ^15^ However, hereditary CRC syndromes only account for a minority of young‐onset CRC cases, and knowledge regarding somatic mutation profiles in early‐onset CRC is limited. Therefore, a comprehensive molecular profile in young CRC patients should be defined, which will help enhance understanding of the unique biology and improve the treatment options for this population.

Recently, next‐generation sequencing (NGS) has led to considerable advances in the analysis of genomic alterations in cancer research and clinical application.^16^, ^17^ In order to better understand the disease biology and determine potentially distinct molecular features for young patients with CRC, the present study comprehensively investigated the molecular signatures from young and old Chinese patients with CRC by NGS, which may provide crucial knowledge regarding the genomics of this population.

## 2 MATERIALS AND METHODS

### 2.1 Patients and sample collection

A total of 75 patients with CRC were enrolled in the present study between September 2017 and May 2019, including 29 young patients (age ≤50 years) and 46 old patients (age >50 years). Tissue samples of these patients were collected for molecular testing. Eligible patients were determined based on the following inclusion criteria: i) Patients were diagnosed with CRC, and ii) ≥150 ng DNA from each tissue sample was successfully extracted.

### 2.2 DNA extraction and library construction

DNA was extracted from tissue samples using the QIAamp Genomic DNA kit (Qiagen GmbH). The quantification and quality of the DNA were assessed using Qubit 2.0 fluorimeter with ds DNA HS assay kit (Thermo Fisher Scientific, Inc.) and the Agilent 2100 BioAnalyzer (Agilent Technologies, Inc.). Sequencing libraries were prepared based on the Illumina standard library construction instructions (Illumina, Inc.).

### 2.3 NGS

The library was hybridized with a targeted panel, including 808 cancer‐related genes, which was enriched for the coding regions and selected introns of genes with known relevance to CRC. Subsequently, the target‐enriched library was sequenced on HiSeq2500 NGS platform (Illumina, Inc.). After the removal of the low‐quality sequencing data, reads were aligned to the human genome reference (hg19) using Burrows‐Wheeler Aligner (BWA, version 0.7.12). PCR duplicates were marked using the MarkDuplicates function in Picard tools (version 2.1.0). Base recalibration was conducted using GATK software (version 3.8). MuTect2 (version 1.1.7) was used to identify single nucleotide variant (SNV) and small insertion or deletion (INDEL), and ≥5 variant supporting reads were required for their filtering. All variants were annotated using ANNOVAR. Copy number variant calling was conducted using CONTRA software (version 2.0.8).

### 2.4 Statistical analysis

The data was analyzed using R (version 3.5.1) and GraphPad Prism (version 5; GraphPad Software, Inc.). Associations between gene mutation status and clinical characteristics were analyzed using Fisher's exact test or *χ* ^2^ test. Correlations between variables were assessed using Pearson's correlation coefficient. Differences in continuous variables were evaluated by Student's t‐test. A two‐sided *p* < 0.05 was considered as statistically significant.

## 3 RESULTS

### 3.1 Patient features

In the present study, a total of 29 young patients (age, ≤50 years) and 46 old patients (age, >50 years) diagnosed with CRC were enrolled. In the young cohort, the median age at diagnosis was 40 years (range, 16–50 years). In the old cohort, the median age at diagnosis was 65 years (range, 51–84 years). There was no difference in the clinical stage between the two groups. Further analysis demonstrated CRC was more likely to occur in the old male patients, although no statistically significant differences were observed (*p* = 0.082). The clinical and pathological characteristics of the patients are listed in Table 1.

**TABLE 1:** Clinical characteristics of young and old patients with colorectal cancer

Characteristics	Total (n = 75)	Young (n = 29)	Old (n = 46)	*p* value
Age, year, median (range)	56 (16–84)	40 (16–50)	65 (51–84)	<0.001
Gender, n (%)				
Male	43 (57.3%)	13 (44.8%)	30 (65.2%)	0.082
Female	32 (42.7%)	16 (55.2%)	16 (34.8%)	
T stage, n (%)				
1	3 (4.0%)	0 (0)	3 (6.6%)	0.131
2	2 (2.7%)	2 (6.9%)	0 (0)	
3	42 (56.0%)	19 (65.5)	23 (50.0%)	
4	20 (26.6%)	6 (20.7%)	14 (30.4%)	
Unknown	8 (10.7%)	2 (6.9%)	6 (13.0%)	
N stage, n (%)				
0	21 (28.0%)	8 (27.6%)	13 (28.3%)	0.151
1	28 (37.3%)	15 (51.7%)	13 (28.3%)	
2	18 (24.0%)	4 (13.8%)	14 (30.4%)	
Unknown	8 (10.7%)	2 (6.9%)	6 (13.0%)	
M stage, n (%)				
0	39 (52.0%)	15 (51.7%)	24 (52.2%)	0.658
1	28 (37.3%)	12 (41.4%)	16 (34.8%)	
Unknown	8 (10.7%)	2 (6.9%)	6 (13.0%)	
Clinical stage, n (%)				
I	2 (2.7%)	0 (0)	2 (4.3%)	0.565
II	13 (17.3%)	4 (13.8%)	9 (19.6%)	
III	24 (32.0%)	11 (37.9%)	13 (28.3%)	
IV	28 (37.3%)	12 (41.4%)	16 (34.8%)	
Unknown	8 (10.7%)	2 (6.9%)	6 (13.0%)	
MMR status, n (%)				
pMMR	68 (90.7%)	26 (89.7%)	42 (91.3%)	0.811
dMMR	7 (9.3%)	3 (10.3%)	4 (8.7%)	

### 3.2 Genomic profile of chinese CRC

**FIGURE 1:**
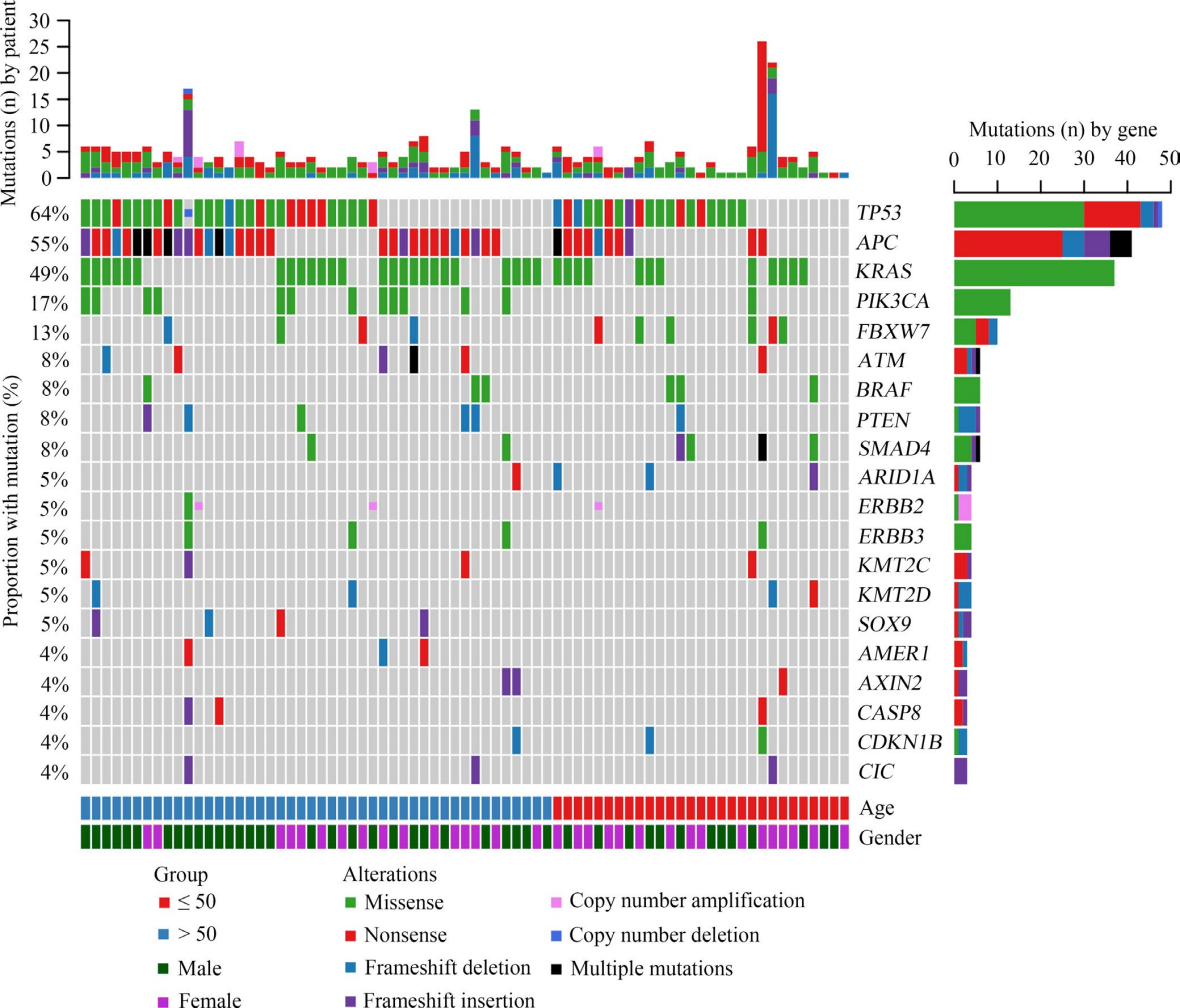
Landscape of genomic alterations among 75 Chinese patients with colorectal cancer (CRC). Genetic mutations were identified by targeted next‐generation sequencing of the tumor tissues of patients with CRC. Abbreviations: Multiple mutations, mutant numbers more than 2

Samples from all 75 patients were profiled by targeted sequencing, and all exhibited at least one genetic alteration. The most commonly mutated genes were *TP53* (64%), *APC* (55%), *KRAS* (49%), *PIK3CA* (17%), and *FBXW7* (13%) (Figure 1). Compared with TCGA data, a significantly higher number of somatic mutations were observed in *CDKN1B* (*p* = 0.029), and significantly fewer mutations in *APC* and *AMER1* were identified among the Chinese CRC patient cohort (*p* = 0.002 and *p* = 0.030, respectively). Furthermore, potentially statistical differences were identified in the frequencies of *PIK3CA* and *SOX9* mutations between TCGA and Chinese cohort (*p* = 0.060 and *p* = 0.087, respectively). Although the *KRAS* mutation rate was higher in the Chinese cohort compared with TCGA cohort, no significant difference was observed (*p* = 0.162) (Table S1).

**FIGURE 2:**
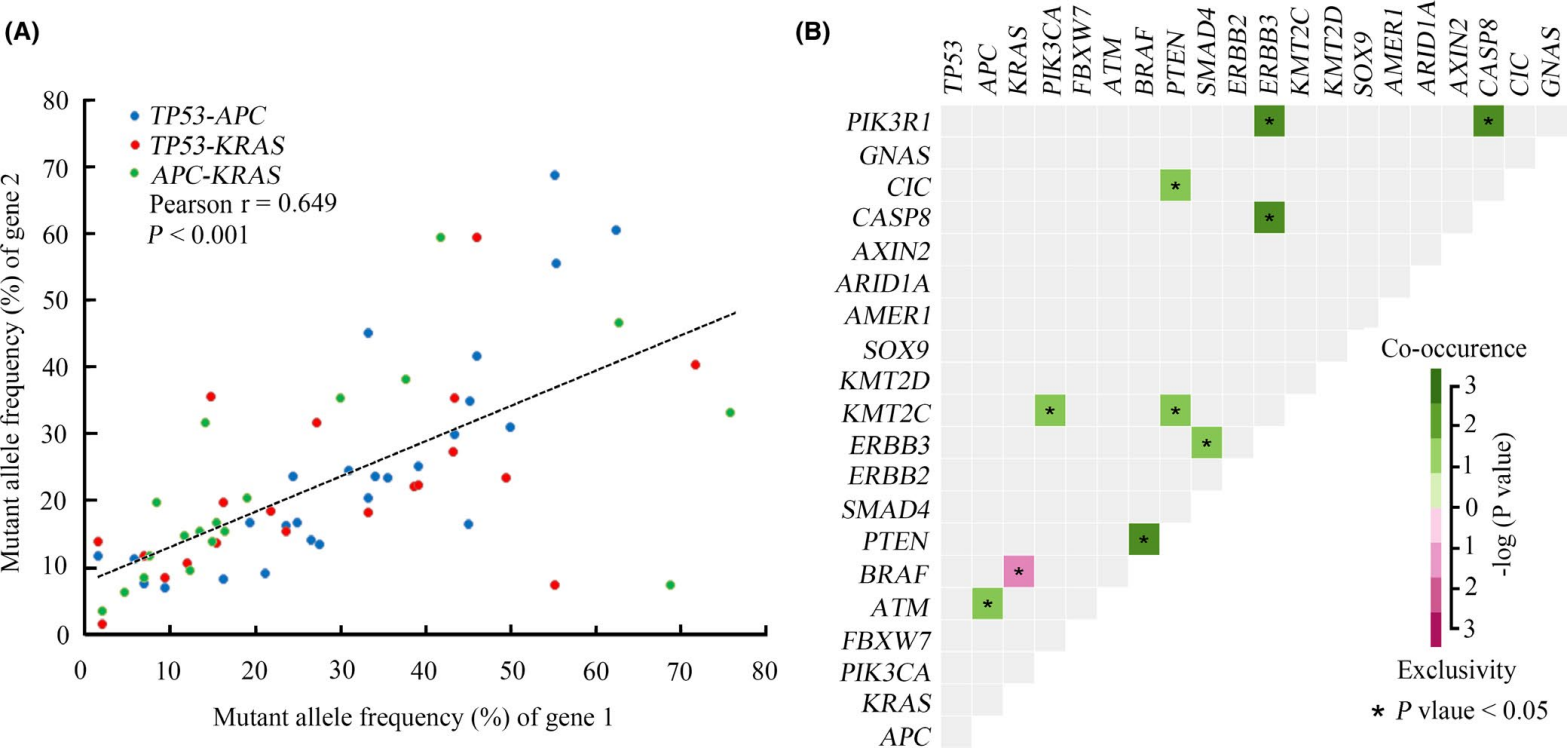
Comprehensive analysis of the associations between genomic mutations of CRC. A, Correlation analysis between mutant allele frequencies in *TP53*, *APC*, and *KRAS*. B, A pairwise association plot for mutant genes in CRC

Among all the patients with CRC, *TP53*, *APC*, and *KRAS* co‐mutations were observed in 10 patients (13.3%), and co‐alterations of *TP53*/*APC*, *TP53*/*KRAS*, and *APC*/*KRAS* were present in 27 (36.0%), 20 (26.7%) and 19 patients (25.3%), respectively. Evolutionary studies of CRC have shown that *TP53*, *APC*, and *KRAS* mutations are the most dominant clonal mutations^18^, ^19^, ^20^; thus, it was hypothesized that there may be a correlation among the mutant allele frequencies of these genes in individual patients. As expected, a significant linear relationship was observed (Figure 2A). Further analysis demonstrated that the mutant types of *TP53*, *APC*, and *KRAS* demonstrated significant differences (Figure S1A), and *TP53* missense mutations or indels tended to coexist with *APC* mutations (Figure S1B). According to the genomic profile, pairwise associations between somatic events in CRC were further investigated. Mutual exclusivity between mutations in *KRAS* and *BRAF*, and co‐mutations of *PTEN* and *BRAF*, *PTEN* and *CIC*, *PIK3R1* and *ERBB3*, *PIK3R1* and *CAPS8*, *PIK3CA* and *KMT2C*, and *APC* and *ATM* were observed (Figure 2B).

### 3.3 Differences in mutation spectrums between young and old CRC groups

**FIGURE 3:**
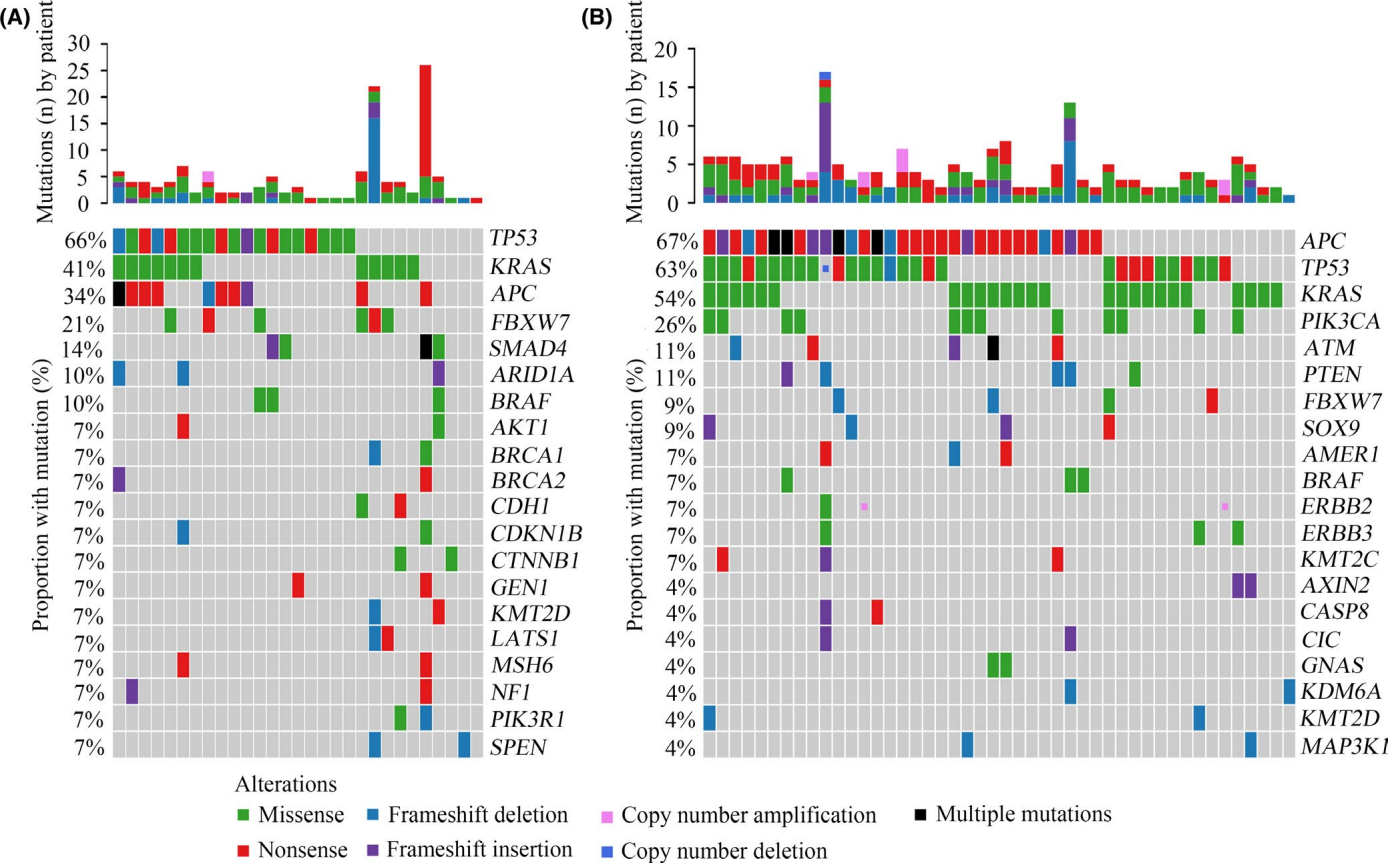
Genomic profiling between young and old patients with CRC. A, Genetic mutations in the young CRC group. B, Genetic mutations in the old CRC group

**FIGURE 4:**
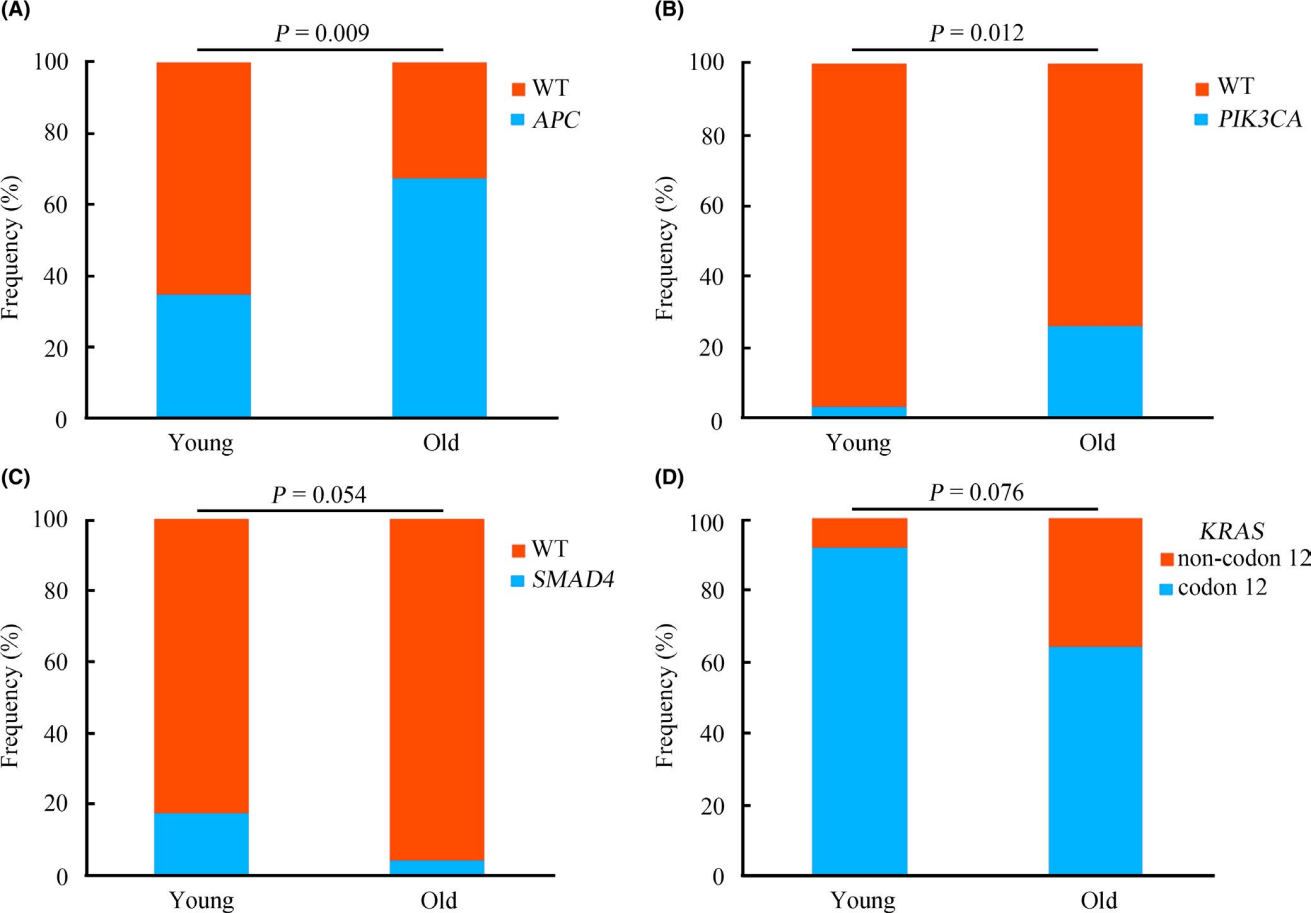
Distributions of representative genomic alterations between young and old groups with CRC. The comparative analysis of frequencies of *APC* (A), *PIK3CA* (B), *SMAD4* (C), and *KRAS* (D) mutations between young and old patients. Abbreviations: WT, wild type

The genomic profiles of young and old groups were compared, and notable differences between them were observed. In the young group, the most commonly mutated genes were *TP53* (66%), *KRAS* (41%), *APC* (34%), *FBXW7* (21%), *SMAD4* (14%), *ARID1A* (10%), and *BRAF* (10%) (Figure 3A). In the old group, the most frequently mutated genes were *APC* (67%), *TP53* (63%), *KRAS* (54%), *PIK3CA* (26%), *ATM* (11%), *PTEN* (11%), *FBXW7* (9%), and *SOX9* (9%) (Figure 3B). Although *TP53*, *APC*, and *KRAS* were the most commonly mutated genes in both two groups, the mutation rates and ranking orders of them were different. Statistical analysis revealed that the frequencies of *APC* and *PIK3CA* mutations in the old group were significantly higher compared with that in the young group (*p* = 0.009 and *p* = 0.012, respectively) (Figure 4A,B). *SMAD4* alterations were potentially associated with young age (*p* = 0.054) (Figure 4C). In addition, *FBXW7* mutations were more likely to occur in young patients, although no statistically significant difference was observed (*p* = 0.137) (Figure S2A). Furthermore, the incidence of *KRAS* mutations between the two groups exhibited no significant difference (*p* = 0.274) (Figure S2B). The mutation loci distributions of *KRAS* were evaluated. In the young group, *KRAS* mutations were mainly distributed in codon 12 (Figure S3A). However, in the old group, *KRAS* mutations were distributed in different codons (Figure S3B). Further analysis indicated that the frequencies of the mutation loci distributions of *KRAS* between the young and old groups exhibited a potential statistical difference (*p* = 0.076) (Figure 4D).

### 3.4 Profiles of clinically actionable mutations

**FIGURE 5:**
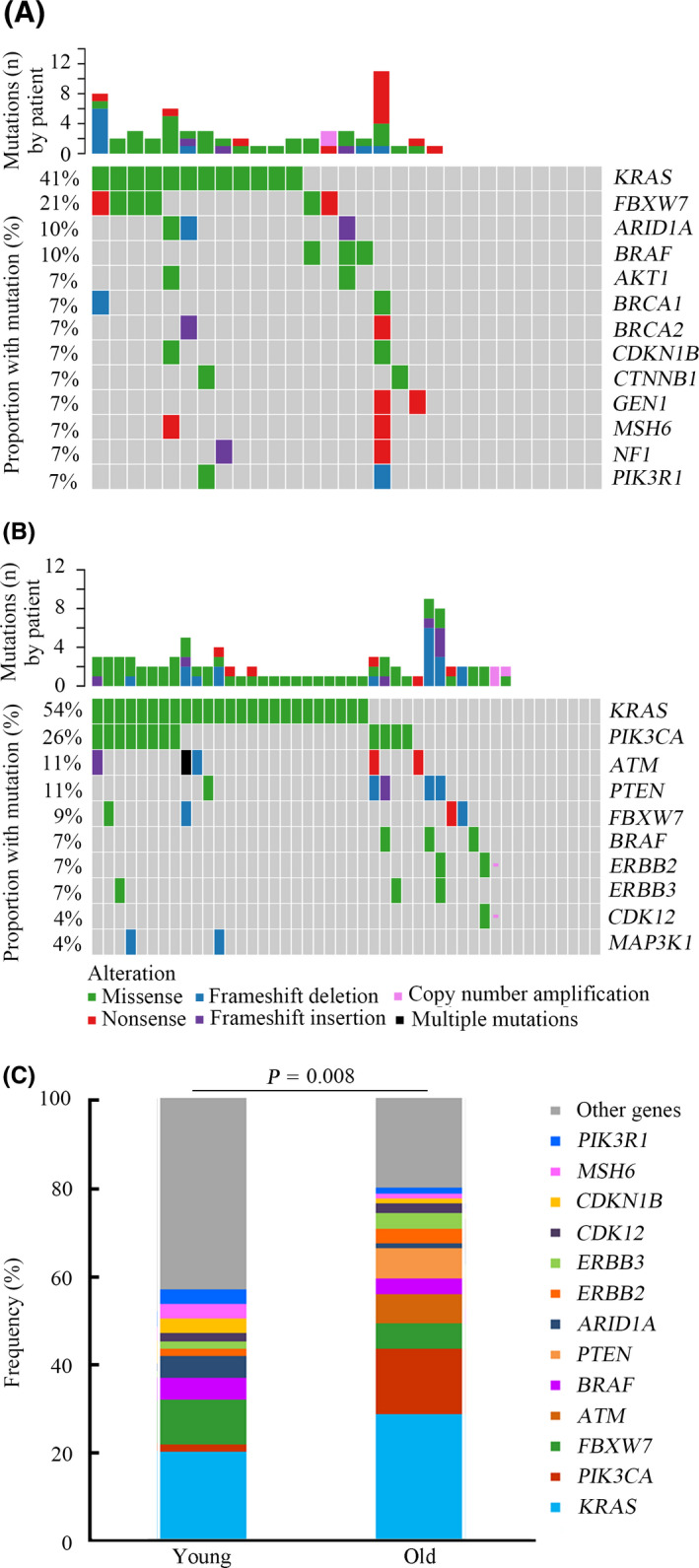
Overview of targeted genomic profiles between young and old groups with CRC. A, The targeted genomic profile in the young patients with CRC. B, The targeted genomic profile in the old patients with CRC. C, The comparative analysis of prevalence of targeted genomic alterations between the young and old patients

In the present study, actionable alterations, defined as molecular targets for new drugs, as well as existing drugs that could guide treatment decisions for patients were evaluated.^21^, ^22^ Overall, 148 actionable mutations were detected from 58 patients (77.3%). Alterations in *KRAS*, *PIK3CA*, *FBXW7*, *ATM*, *BRAF*, and *PTEN* were the most common targets observed in the present study (Figure S4). Among all the patients, 69.0% of young cases harbored at least one actionable alteration. The most common actionable mutations in this group occurred in the following: *KRAS* (41%), *FBXW7* (21%), *ARID1A* (10%), *AKT1* (10%), and *BRAF* (10%) (Figure 5A). Additionally, 82.6% of old patients harbored at least one actionable alteration, and the most common actionable mutations occurred in the following: *KRAS* (54%), *PIK3CA* (26%), *PTEN* (11%), and *ATM* (11%) (Figure 5B). Based on all the actionable mutations identified, further analysis revealed a significant difference between the young and old groups regarding the targeted genomic profile (*p* = 0.008) (Figure 5C).

**FIGURE 6:**
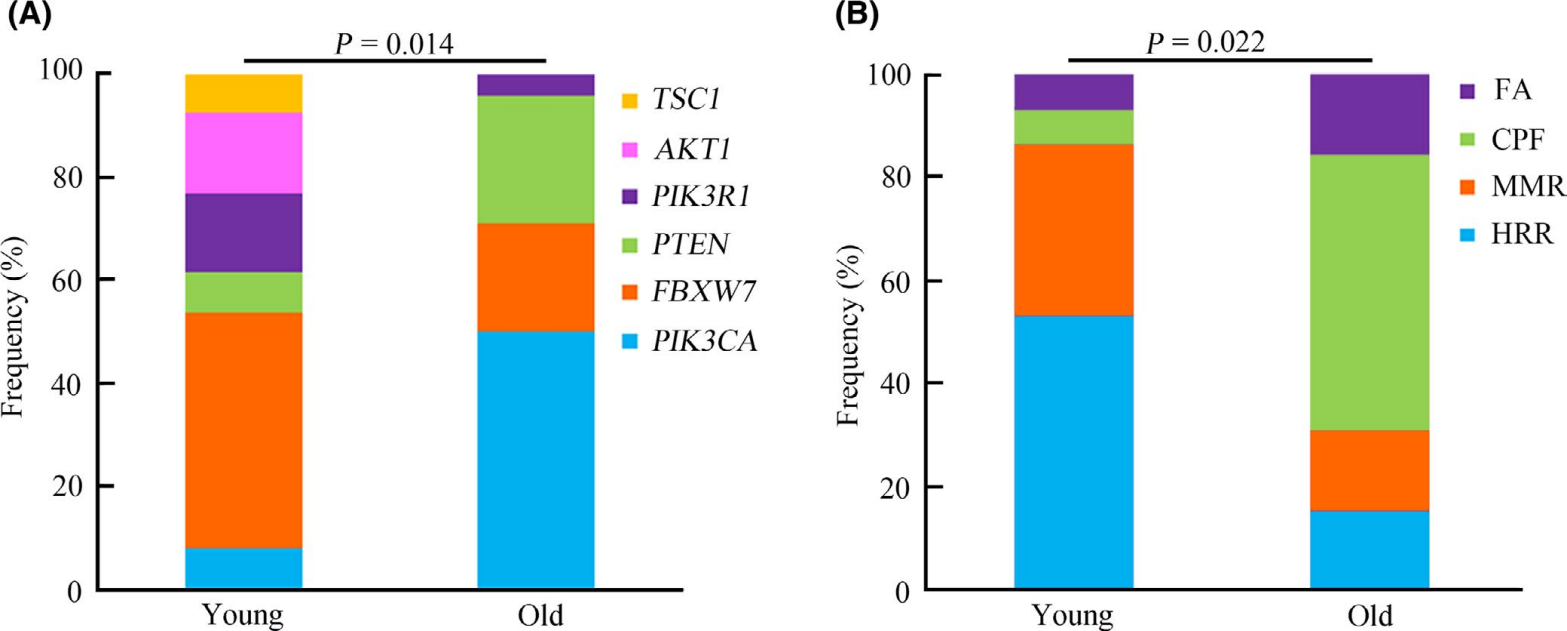
Comprehensive analysis of the composition of PI3K and DDR signaling pathways between young and old groups with CRC. A, Comparative analysis of the composition of the PI3K signaling pathway between young and old patients. B, Comparative analysis of the composition of the DDR signaling pathway between young and old patients

Pathway enrichment analysis was next performed according to the actionable mutations, to investigate functionally aberrant pathways. The most enriched pathways associated with actionable mutations in CRC included the MAPK (34.5%), PI3K (25.7%), and DDR (DNA damage repair) (18.9%) signaling pathways. Additionally, the ERBB, cell cycle, chromatin remodeling, and Wnt signaling pathways were also observed to be associated (Figure S5A). The frequencies of different pathways were then compared between the young and old groups, which revealed no significant difference between the two groups (*p* = 0.527) (Figure S5B). Notably, the proportions of mutated genes in the PI3K signaling pathway were significantly different between the two groups (*p* = 0.014) (Figure 6A). Previous work has reported that the DDR system comprises different pathways, including mismatch repair (MMR), base excision repair (BER), check point factors (CPF), Fanconi anemia (FA), homologous recombination repair (HRR), nucleotide excision repair (NER), nonhomologous end‐joining (NHEJ), and DNA translesion synthesis (TLS).^23^, ^24^ The composition of the DDR system between the two groups was then further analyzed, which demonstrated that HRR, MMR, CPF, and FA were the most common DDR pathways in CRC. Among these, HRR and MMR mainly occurred in the young group; however, CPF was more likely to occur in the old group (*p* = 0.022) (Figure 6B).

### 3.5 Tumor mutation burden analysis

**FIGURE 7:**
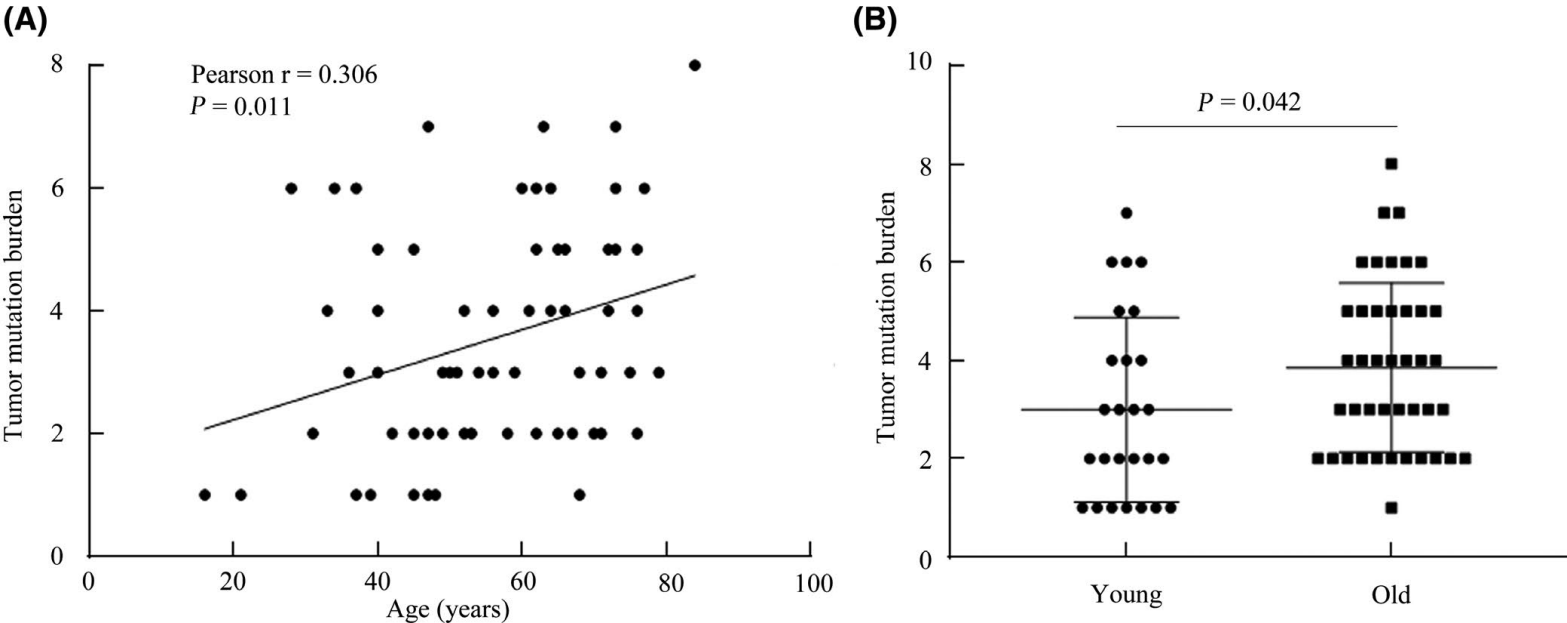
Association between TMB and age in CRC. A, Correlation analysis between TMB and age in pMMR patients with CRC. B, Comparison of TMB between young and old patients with CRC. Abbreviations: TMB, tumor mutation burden; pMMR, DNA mismatch repair‐proficient

**FIGURE 8:**
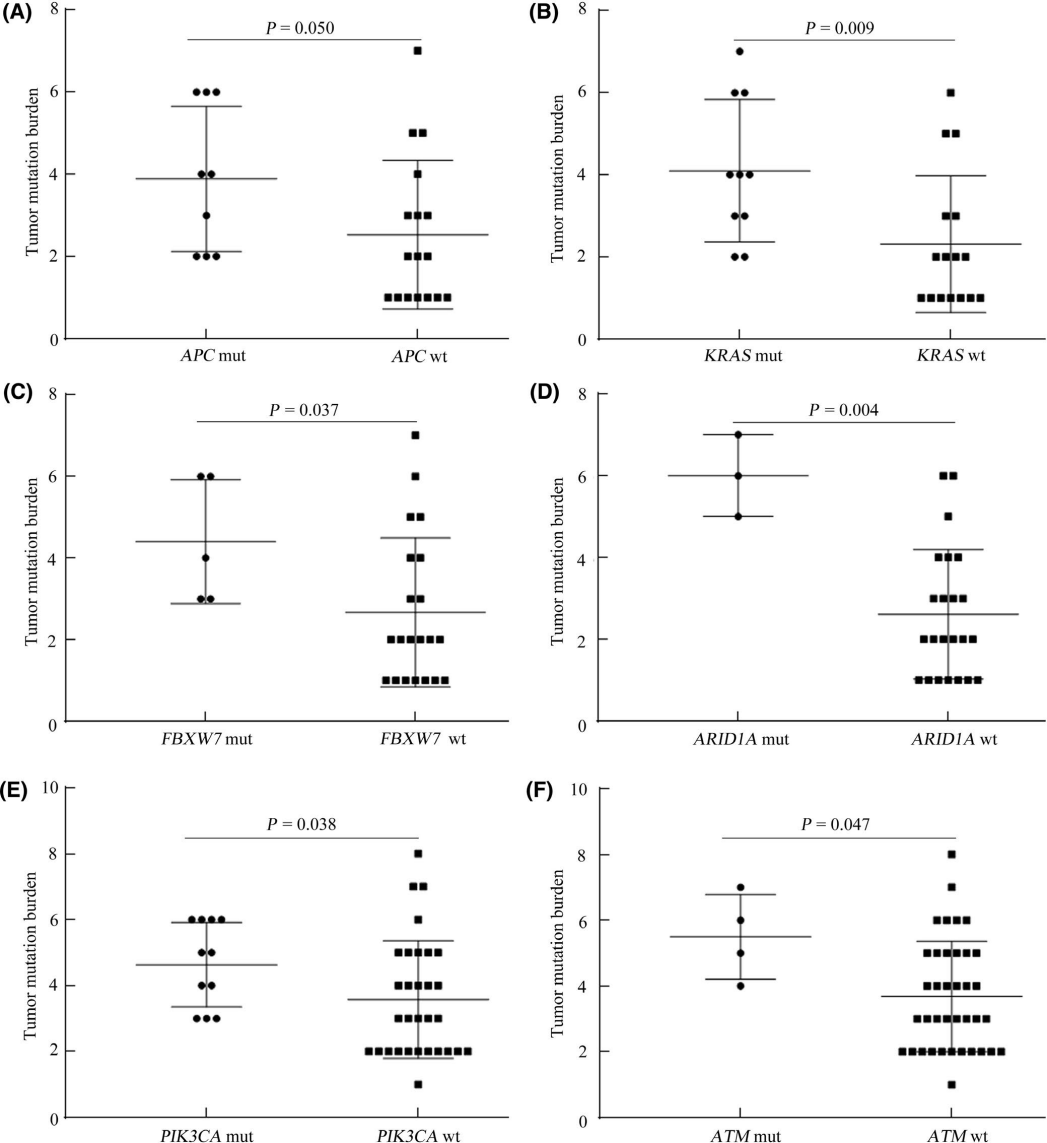
Relationship between TMB and certain specific gene mutations in pMMR patients with CRC. A‐D, Comparison of TMB between patients with and without *APC*, *KRAS*, *FBXW7*, and *ARID1A* in young patients with CRC. E and F, Comparison of TMB between patients with and without *PIK3CA* and *ATM* in old patients with CRC

Numerous studies have indicated that TMB is a potential biomarker to predict the efficacy of immune‐checkpoint inhibitors in various cancer types.^25^, ^26^, ^27^ In advanced CRC, dMMR/MSI‐H is the standard marker for immunotherapy. Although previous work has reported dMMR/MSI‐H was dramatically correlated with a high TMB,^28^ it is not known whether TMB is associated with other factors, except for dMMR/MSI‐H, in CRC. In the present study, the TMB was calculated using with the total number of nonsynonymous alterations (single‐nucleotide variants and small insertions or deletions) for each patient.^29^ The TMB in patients with and without dMMR was then compared. As expected, patients with dMMR exhibited a significantly higher TMB compared with patients with pMMR (*p* = 0.002) (Figure S6). Among patients with pMMR, the association between TMB and age was investigated, and a significantly linear relationship was observed (Pearson *r* = 0.306, *p* = 0.011) (Figure 7A). Furthermore, the TMB in the old group was remarkably higher compared with that in the young group (*p* = 0.042) (Figure 7B). In CRC, the association between genomic mutations and TMB is not clear. Therefore, the present study further analyzed the influence of gene mutations on TMB among young and old patients with pMMR CRC. In this study, young patients with *APC*, *KRAS*, *FBXW7*, and *ARID1A* mutations were more likely to exhibit a high TMB (*p* < 0.05) (Figure 8A–D). In contrast, old patients with *PIK3CA* and *ATM* mutations harbored strikingly high TMB (*p* < 0.05) (Figure 8E, F).

## 4 DISCUSSION

An increasing incidence of CRC in young patients has attracted attention from the public, and caused a heavy burden on patients and societies. Although a number of studies have indicated that CRC in the young population harbors unique clinical features,^5^, ^6^, ^7^, ^8^ the genomic signatures of this population are not well understood. Based on the comprehensive analysis of young and old Chinese patients with CRC, the present study provides novel insights into the differences of genomic profiles between the two groups.

The present study observed the differences in the prevalence of several driver gene mutations between Chinese and TCGA cohorts, indicating a specific mutational spectrum in Chinese patients with CRC. The majority of CRC patients carried mutations in two genes of *TP53*/*APC*/*KRAS*, mutant allele frequencies of which displayed a significant linear correlation, confirming that mutations in these genes regularly co‑occur as clonal events in the evolution of CRC. The patterns of co‐mutation and mutual exclusivity were further explored, which indicated that the PI3K signaling pathway tends to co‐exist with another signaling pathway in CRC.

The genomic landscape in the young group exhibited notable differences from that of the old group. Significantly, a higher prevalence of *APC* and *PIK3CA* mutations were identified in old patients, which was consistent with a previous study.^30^ Additionally, *SMAD4* mutation was more likely to occur in the young group. Numerous studies reported that *SMAD4* mutation tends to be a higher risk feature associated with poor prognosis in CRC.^31^, ^32^, ^33^ According to previous studies, young patients with CRC exhibited a shorter survival time.^9^, ^10^ The present data provided a novel insight into the differences in prognosis between young and old CRC groups from a molecular perspective. A previous study has reported that *KRAS* mutations were more commonly observed in old patients with CRC.^30^ In the current study, no differences in *KRAS* mutation frequencies between the two groups were observed, which may be attributed to the difference in race. However, based on the mutation loci distributions of *KRAS*, it was identified that young patients predominantly carried *KRAS* codon 12 mutations. By contrast, *KRAS* mutations in old patients tended to be distributed in different loci. A number of studies have demonstrated that mutations in *KRAS* codon 12 are associated with poor survival in CRC.^34^, ^35^ Based on the present results, the distribution of *KRAS* mutations may further help explain the difference in prognosis in young and old CRC patients.

Targeted therapies and immunotherapies have markedly improved the clinical outcome for certain patients with advanced CRC. However, limited understanding of CRC‐related molecular biomarkers and indicated limitations of certain anticancer drugs hinders the development of such therapies and corresponding clinical trial design. Therefore, comprehensive analysis of clinically actionable mutations that could potentially guide therapeutic decisions is essential to promote the treatment of patients with CRC. The present results revealed 77.3% of patients carried at least one clinically actionable alteration, which indicates the importance of comprehensive molecular testing and the necessity of “basket” clinical trials in which patients can enroll according to their actionable alterations. In addition, although no difference was observed in pathways related to actionable mutations between young and old groups, the molecular compositions of some pathways were different between them, further demonstrating the significance of comprehensive elaborate genetic testing and personalized therapy.

Currently, the clinical association of MSI‐H/dMMR has been highlighted by the fact that it is an effective predictive biomarker for the efficacy of immune checkpoint inhibitors.^36^, ^37^ In advanced CRC with MSI‐H/dMMR, given the encouraging effect of immunotherapies, PD‐1 inhibitors have been approved as first line therapy and beyond by the current National Comprehensive Cancer Network guidelines. However, MSI‐H only occurs in approximately 15% of CRC cases.^38^ Therefore, there is an urgent need for additional biomarkers that can identify patients likely to respond to PD‐1/PD‐L1 inhibition. Multiple studies have revealed that the TMB is a potential biomarker to predict the response to immune‐checkpoint inhibitors.^25^, ^26^, ^27^ Owing to the defect of the MMR system, MSI‐H/dMMR tumors tend to harbor a high TMB, whereas not every CRC with high TMB exhibits MSI‐H/dMMR signature. Therefore, a full understanding of TMB and its correlation with other factors is of great significance to guide personalized therapy and promote the clinical management of patients with CRC. Notably, the overall TMB in the pMMR CRC cohort increased with increasing age, and similar results have been observed in other cancer types.^39^ Previous reports investigating the mutation profile within certain cancers have demonstrated a positive relationship between the age of cancer diagnosis and the mutant numbers, indicating that the mutation process may occur at a constant rate over time and eventually lead to an increased mutational burden.^40^, ^41^, ^42^ Furthermore, the present study also comprehensively evaluated the correlation between TMB and specific genetic mutations in young and old patients with pMMR CRC. We found that somatic mutations associated with high TMB between the two groups showed some differences. For instance, *APC* and *KRAS* mutations were observed to be associated with high TMB in young patients, whereas *PIK3CA* mutations were associated with high TMB in old patients. Currently, no significant associations between these frequently mutated genes and high TMB were found in CRC. Additionally, *ARID1A* and *ATM* alterations classified as DDR mutations were related to high TMB in young and old patients, respectively. Although numerous studies have revealed the correlation between DDR alterations and high TMB in CRC,^43^, ^44^ our results further confirmed these findings and revealed different mutations in DDR genes associated with high TMB between young and old tumors. The findings in the present study recognized unique molecular features in future immune therapy trials for pMMR CRC, and highlighted the importance of personalized immunotherapy in this population. Therefore, these data may provide more possibilities for developing novel biomarkers that could predict the efficacy of immune checkpoint inhibitor therapies in CRC and further implied that the biomarkers need to be considered differently between young and old groups.

## 5 CONCLUSIONS

To the best of our knowledge, this is the first NGS study to compare genomic profiles between young and old Chinese cohorts with CRC. The results demonstrated that young patients with CRC presented with some unique molecular features. In addition, the comprehensive investigation of clinically actionable mutations and TMB will contribute to the improvement of personalized therapy and clinical management in CRC.

## ETHICS APPROVAL AND CONSENT TO PARTICIPATE

All participants provided written informed consent before sample collection. This study was approved by the ethical committee of the Affiliated Sir Run Run Shaw Hospital of Zhejiang University School of Medicine.

## CONFLICT OF INTEREST

H.N.W, L.F, and S.B.C are employees of Acornmed Biotechnology Co., Ltd. The other authors have declared that no competing interest exists.

## AUTHOR CONTRIBUTIONS

S.D, F.W, H.Q.C, and X.Z designed the study. L.S, B.J.B, and K.K.C enrolled the patients and collected the corresponding clinical information. H.N.W, L.F, and S.B.C conducted the data analysis. F.W, H.Q.C, and X.Z wrote the paper. Other authors discussed and commented on the manuscript. All authors read and approved the final manuscript.

## Supporting information

Supplementary Material

## Data Availability

The data that support the findings of this study are available from the corresponding author upon reasonable request.
