# Supplementary material for: Comparative genomic signatures in young and old Chinese patients with colorectal cancer
Source: Cancer Med. 2021 May 26;10(13):4375–86. doi: 10.1002/cam4.3987 (PMC8267122; doi:10.1002/cam4.3987)
Supplement: Supplementary file 1 — Supplementary Material [file CAM4-10-4375-s001.docx]

**Supplementary Figures and Tables**

**
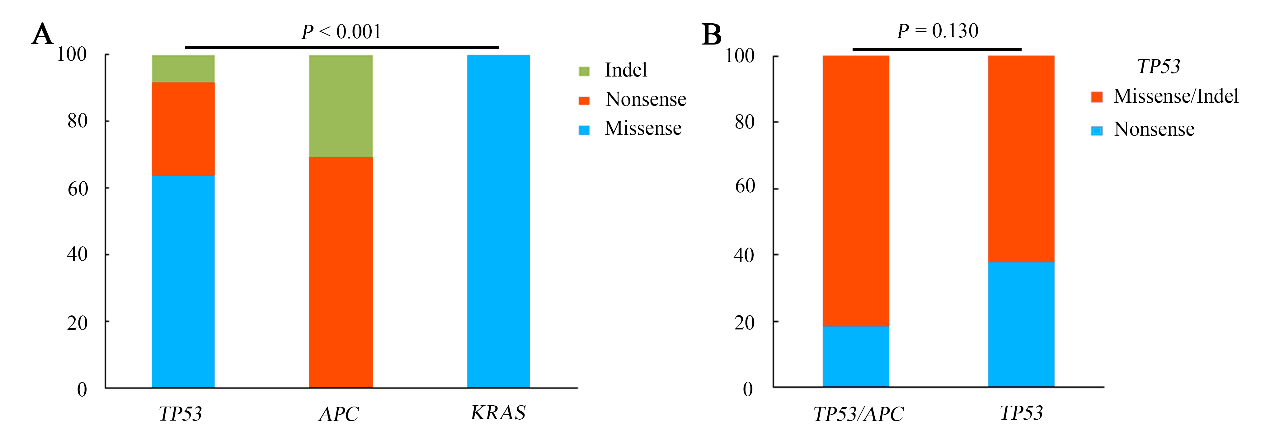
**

**Figure S1.** Analysis of mutation patterns in *TP53*, *APC*, and *KRAS*. A, Analysis of mutation types of *TP53*, *APC*, and *KRAS*. B, Distribution of mutation types of *TP53* mutations in *TP53*-mutated CRC patients with and without *APC* mutations. Abbreviations: Indel, small insertion or deletion.

**
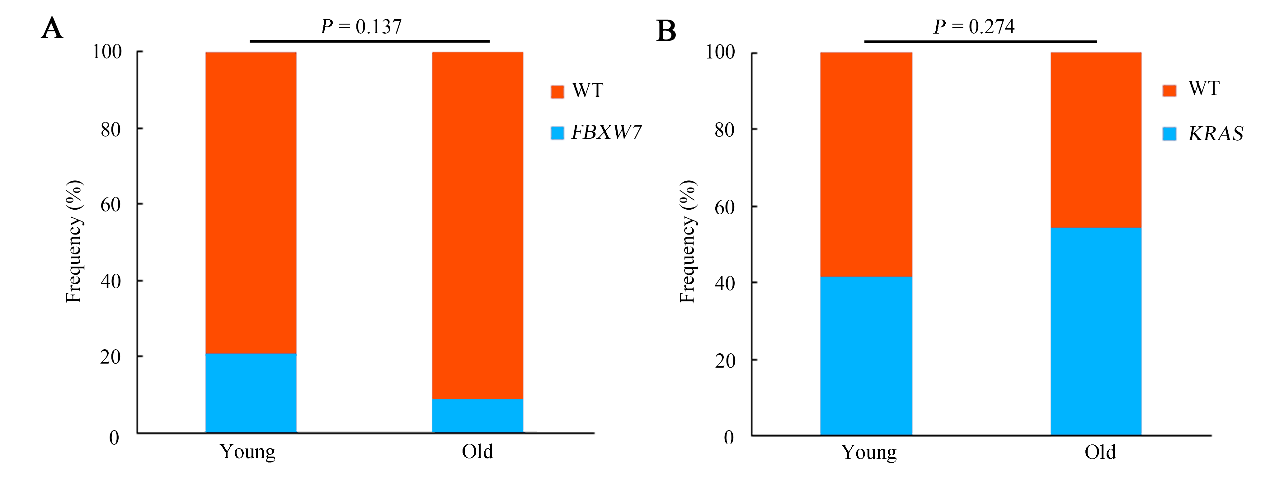
**

**Figure S2.** Distributions of representative genetic mutations between young and old groups with CRC. The comparative analysis of *FBXW7* (A) and *KRAS* mutations (B) between young and old patients. Abbreviations: WT, wild type.

**
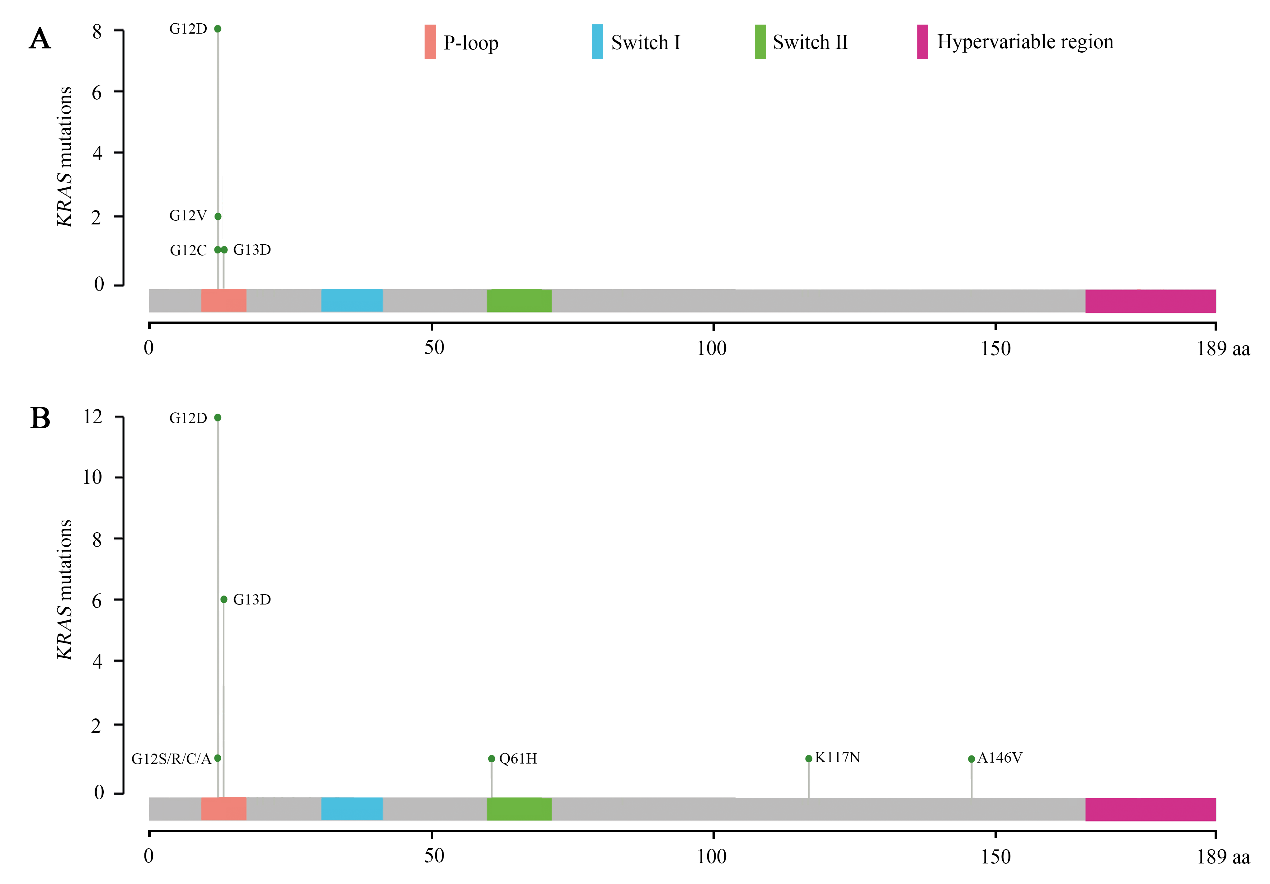
**

**Figure S3.** The localization of *KRAS* mutations in functional domains between young and old groups with CRC. A, Distribution of *KRAS* mutations identified in young patients with CRC. B, Distribution of *KRAS* mutations identified in old patients with CRC.

**
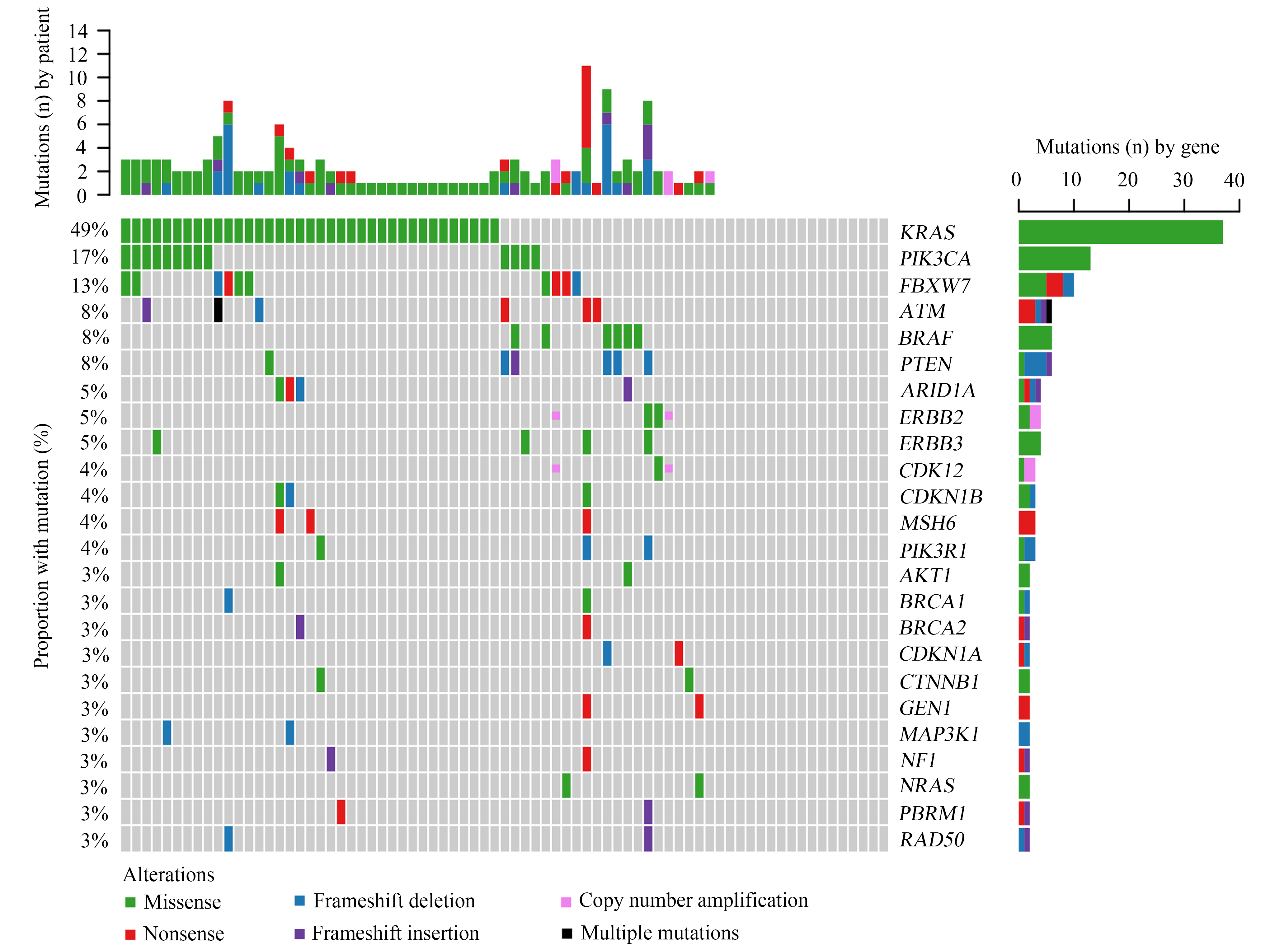
**

**Figure S4.** Landscape of targetable alterations among 75 Chinese patients with CRC. Targetable mutations were identified by targeted next-generation sequencing in the tumor tissues of patients. Abbreviations: Multiple mutations, mutant numbers more than 2.

**
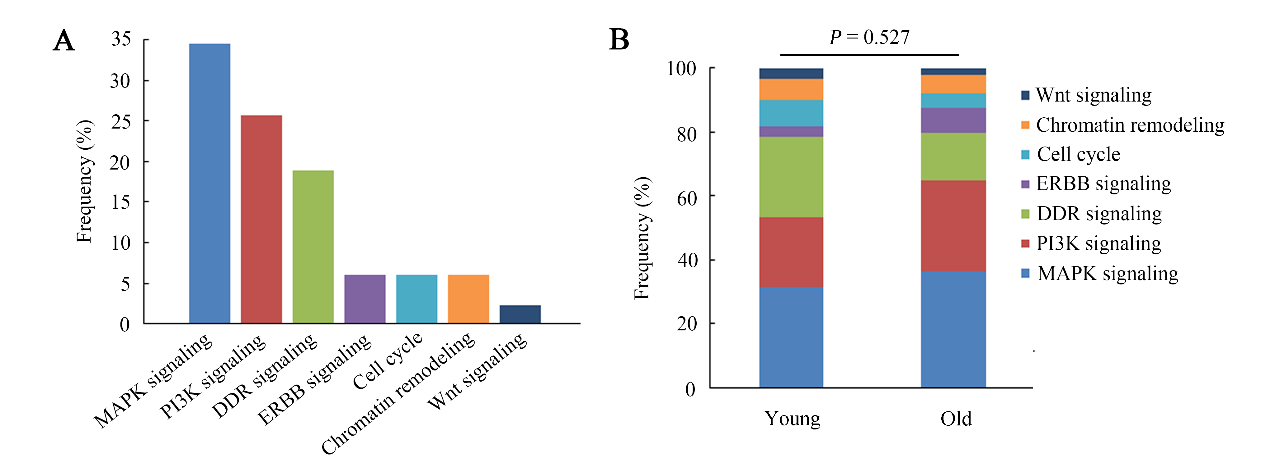
**

**Figure S5.** Analysis of targeted alterations involved in signaling pathways in patients with CRC. A, Frequencies of targeted signaling pathways in CRC. B, Comparative analysis of the prevalence of targeted signaling pathways between young and old patients with CRC.

**
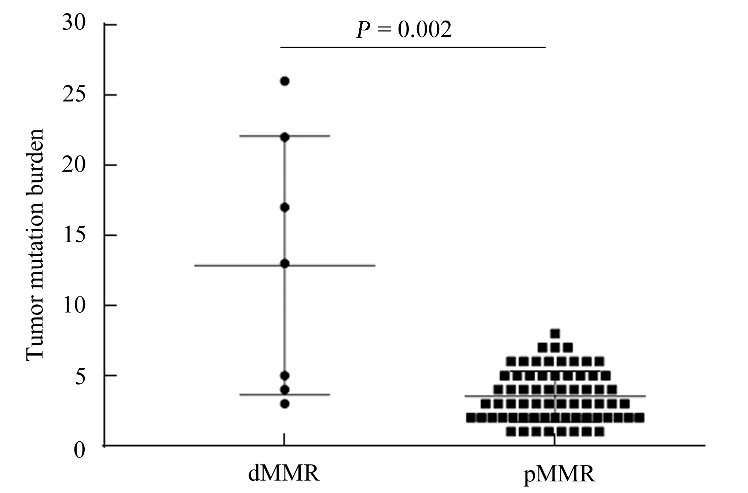
**

**Figure S6.** Comparative analysis of the TMB between dMMR and pMMR patients with CRC. Abbreviations: dMMR, DNA mismatch repair-deficient.

**Table S1.** Frequencies of somatic mutations among patients with colorectal cancer in the present cohort and TCGA cohort.

| Somatic mutations | Frequency, (%) | | *P* value |
| --- | --- | --- | --- |
|  | TCGA cohort (n = 534) | The present cohort (n = 75) |  |
| *TP53* | 58.8% | 64.0% | 0.391 |
| *APC* | 72.5% | 54.7% | 0.002 |
| *KRAS* | 40.8% | 49.3% | 0.162 |
| *PIK3CA* | 27.5% | 17.3% | 0.060 |
| *FBXW7* | 16.9% | 13.3% | 0.441 |
| *ATM* | 13.1% | 8.0% | 0.210 |
| *BRAF* | 11.8% | 8.0% | 0.331 |
| *PTEN* | 6.4% | 8.0% | 0.593 |
| *SMAD4* | 12.9% | 8.0% | 0.225 |
| *ARID1A* | 10.9% | 5.3% | 0.138 |
| *ERBB2* | 3.9% | 5.3% | 0.567 |
| *ERBB3* | 5.4% | 5.3% | 0.972 |
| *KMT2C* | 10.5% | 5.3% | 0.161 |
| *KMT2D* | 11.6% | 5.3% | 0.102 |
| *SOX9* | 12.0% | 5.3% | 0.087 |
| *AMER1* | 12.5% | 4.0% | 0.030 |
| *AXIN2* | 5.4% | 4.0% | 0.603 |
| *CASP8* | 3.9% | 4.0% | 0.978 |
| *CDKN1B* | 0.9% | 4.0% | 0.029 |
| *CIC* | 6.0% | 4.0% | 0.488 |
